# Multiple Mechanisms are Involved in Salt-Sensitive Hypertension-Induced Renal Injury and Interstitial Fibrosis

**DOI:** 10.1038/srep45952

**Published:** 2017-04-06

**Authors:** Shi-Yao Wei, Yu-Xiao Wang, Qing-Fang Zhang, Shi-Lei Zhao, Tian-Tian Diao, Jian-Si Li, Wen-Rui Qi, Yi-Xin He, Xin-Yu Guo, Man-Zhu Zhang, Jian-Yu Chen, Xiao-Ting Wang, Qiu-Ju Wei, Yu Wang, Bing Li

**Affiliations:** 1 Department of Nephrology, Second Affiliated Hospital of Harbin Medical University, Harbin, People’s Republic of China; 2 Financial Mathematics, Beijing Normal University-Hong Kong Baptist University United International College Zhuhai, People’s Republic of China.

## Abstract

Salt-sensitive hypertension (SSHT) leads to kidney interstitial fibrosis. However, the potential mechanisms leading to renal fibrosis have not been well investigated. In present study, Dahl salt-sensitive (DS) rats were divided into three groups: normal salt diet (DSN), high salt diet (DSH) and high salt diet treated with hydrochlorothiazide (HCTZ) (DSH + HCTZ). A significant increase in systolic blood pressure (SBP) was observed 3 weeks after initiating the high salt diet, and marked histological alterations were observed in DSH rats. DSH rats showed obvious podocyte injury, peritubular capillary (PTC) loss, macrophage infiltration, and changes in apoptosis and cell proliferation. Moreover, Wnt/β-catenin signaling was significantly activated in DSH rats. However, HCTZ administration attenuated these changes with decreased SBP. In addition, increased renal and urinary Wnt4 expression was detected with time in DSH rats and was closely correlated with histopathological alterations. Furthermore, these alterations were also confirmed by clinical study. In conclusion, the present study provides novel insight into the mechanisms related to PTC loss, macrophage infiltration and Wnt/β-catenin signaling in SSHT-induced renal injury and fibrosis. Therefore, multi-target therapeutic strategies may be the most effective in preventing these pathological processes. Moreover, urinary Wnt4 may be a noninvasive biomarker for monitoring renal injury after hypertension.

Hypertension is a chronic disease with an increasing worldwide prevalence. Clinical investigations have shown that sodium reduction is associated with increased systolic blood pressure (SBP) in both normotensive and hypertensive individuals^1^. This phenomenon has been defined as “salt sensitivity”^2^. Renal fibrosis is characteristic of salt-sensitive hypertension (SSHT), and Dahl salt-sensitive (DS) rats are widely used to study SSHT^3^. The underlying mechanisms by which SSHT causes renal fibrosis have not been sufficiently elucidated. Furthermore, Western and Japanese guidelines recommend diuretics as the first-line treatment for hypertension^4^,^5^. Because these types of antihypertensive drugs are often used in combination in clinical practice, we used hydrochlorothiazide (HCTZ) treatment to better understand the underlying mechanisms of SSHT-induced renal fibrosis.

DS rats that consume a high salt diet develop SSHT and chronic ischemic nephropathy, which has been demonstrated to induce renal inflammation, tubular atrophy, and interstitial fibrosis, eventually leading to renal dysfunction^3^. In our previous studies, we demonstrated that peritubular capillary (PTC) loss promoted the development of fibrosis, although the effective intervention could ameliorate renal function^6^,^7^. The injury to and loss of PTCs result in impaired oxygen and nutrient delivery to tubules and interstitial cells, which is correlated with tubular atrophy and fibrosis. Furthermore, PTC loss is correlated with renal impairment and interstitial inflammation in animal models and in patients with native kidney disease^8^,^9^. Thus, we questioned whether PTC loss is related to chronic SSHT-induced renal failure. We also hypothesized that early PTC loss potentially underlies the progression of SSHT-induced renal dysfunction.

Macrophages play important roles in health and disease, with functions including immune surveillance, bacterial killing, tissue remodeling and repair, and the clearance of cell debris^10^. Macrophages have both beneficial and detrimental effects on the outcome of several diseases, depending on the cellular activation state and the microenvironment^11^. Our previous studies provided evidence that macrophages play an active role in regeneration and repair through canonical Wnt/β-catenin signaling during kidney injury. Furthermore, autophagic proteins can be recruited by macrophages to clear phagocytosed apoptotic cells and other debris^12^,^13^. Certainly, there is vast conclusive evidence that the accumulation of macrophages is correlated with renal dysfunction in fibrosis models, such as the UUO and STNx models^14^,^15^. In the present study, we aimed to study the role of macrophages in the development of SSHT.

The canonical Wnt/β-catenin pathway, a highly conserved pathway in multicellular organisms, is known to play a critical role in nephrogenesis in the adult kidney^16^. Wnt signaling, however, appears to be silenced and then reactivated in diverse adult kidney diseases, including ischemia/reperfusion injury, glomerular diseases, diabetic nephropathy, obstructive nephropathy, and polycystic kidney disease^17^,^18^. Consistent with these observations, changes in Wnt ligands are associated with activation of the Wnt/β-catenin pathway. Wnt4, a Wnt signaling ligand, has been reported to be upregulated after renal epithelial injury in models of renal fibrosis and to disrupt renal tubular epithelial structures^19^,^20^. In the present study, we investigated whether Wnt/β-catenin signaling and the activation of Wnt ligands are associated with the development of SSHT-induced chronic kidney disease.

## Results

### Systolic blood pressure, renal resistive index (RI), and urinary and serum biochemical analysis

**Figure 1:**
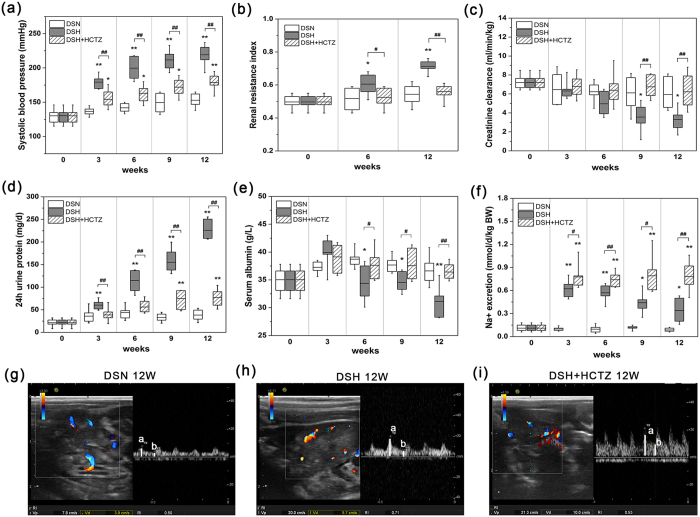
Systolic blood pressure, renal ultrasound analysis, renal function, proteinuria, and sodium metabolism in DS rats. (**a**) SBP was elevated in DS rats fed the 8% salt diet compared with those fed the 0.3% salt diet or those treated with HCTZ. (**b**) The graph represents the renal RI. The RI was calculated as (**a**,**b**)/a, where “a” is the peak systolic velocity, and “b” is the end diastolic velocity. (**c**) Creatinine clearance decreased beginning at week nine on a high salt diet. (**d**) The high salt diet increased protein excretion starting at week three. (**e**) At 6 weeks, serum albumin was significantly decreased in the DSH group compared with the DSN group, and HCTZ treatment improved this change. (**f**) The graph shows Na^+^ excretion in urine, which was increased by HCTZ treatment. (**g**–**i**) Representative images of the renal resistive index (RI) measurements for each group. Pulsed Doppler ultrasounds were obtained for the intra-renal artery of the renal cortex. *p < 0.05, **p < 0.01 versus DSN group. ^#^p < 0.05, ^##^p < 0.01: DSH + HCTZ group versus DSH group.

Compared with the DSN group, DSH rats showed elevated SBP after three weeks on a high salt diet, which continued to increase in response to this diet (Fig. 1a). HCTZ treatment significantly prevented the increase in SBP induced by the high salt diet (Fig. 1a). A renal ultrasound was performed to detect changes in the renal RI (Fig. 1b, g–i). In parallel with the alterations in SBP, the renal RI increased significantly in the DSH group (Fig. 1g) compared with the DSN group (Fig. 1h). The increased RI was attenuated in DSH rats after HCTZ administration (Fig. 1i). These results are expressed graphically in Fig. 1b.

Due to the high salt diet, DSH rats not only developed severe hypertension (HTN) but also displayed a gradual decline in renal function. As shown in Fig. 1c, creatinine clearance at 9 weeks was markedly lower in DSH rats compared with DSN rats. Furthermore, urinary protein excretion was considerably increased at 3 weeks in DSH rats but remained unaltered in DSN rats (Fig. 1d). Serum albumin levels were decreased after 6 weeks of salt loading compared with the DSN group (Fig. 1e). HCTZ treatment significantly ameliorated these changes (Fig. 1c–e). Additionally, urinary sodium excretion was higher in the DSH group compared with the DSN group and further increased after HCTZ administration (Fig. 1f).

### Tubular injury and renal interstitial fibrosis in SSHT

**Figure 2:**
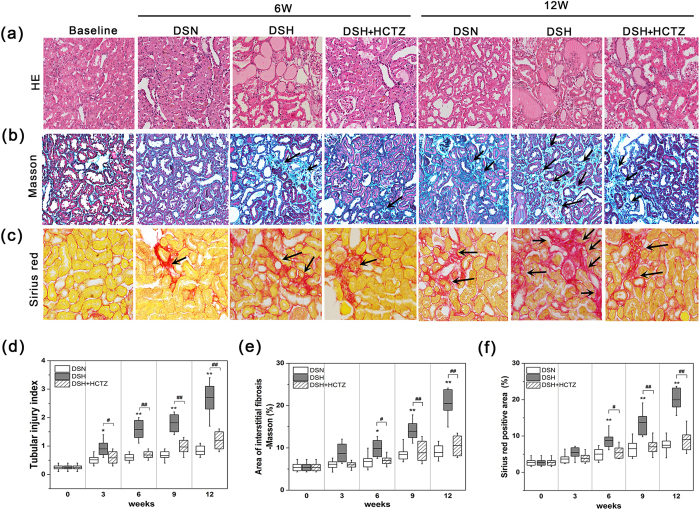
High salt loading induces tubular injury and renal interstitial fibrosis in DS rats. (**a**) Representative light microscopy images of hematoxylin-eosin (HE)-stained sections of the renal cortex from each group (magnification, 200X; scale bar = 50 μm). (**b**) Representative photomicrographs of Masson’s trichrome staining of cortical kidney sections from each group. Fibrosis (black arrows) and fibrotic areas are depicted in the renal cortical sections (magnification, 200X). (**c**) Representative micrographs of Sirius red-stained renal cortex sections, showing fibrosis (black arrows) and fibrotic areas (magnification, 200X). (**d**) Graph of the semi-quantitative determination of tubular injury lesions by HE staining. (**e**) Diagram of the area of Masson staining with renal cortex fibrosis per high-power field (HPF) at each time point. (**f**) Diagram of the area of Sirius red-positive staining per HPF; this parameter was used to evaluate the fibrotic area in the renal cortex in the long-term experiment. *p < 0.05, **p < 0.01 versus DSN group. ^#^p < 0.05, ^##^p < 0.01: DSH + HCTZ group versus DSH group.

Compared with the DSN group, significant tubular injury, as evidenced by vacuolation and desquamation of renal epithelial cells, intratubular proteinaceous cast formation and inflammatory cell infiltration, was observed at week 3 in DSH rats, and these pathologic alterations worsened over time. However, HCTZ treatment significantly attenuated these changes (Fig. 2a). These results were validated by semiquantitative histopathological analysis (Fig. 2d). Renal fibrosis was assessed by Masson’s trichrome staining and Sirius red staining (Fig. 2b,c). At week 6, renal fibrosis was evident in the DSH group by Masson’s trichrome staining (Fig. 2b) and Sirius red staining (Fig. 2c) compared with the DSN group. However, these changes were strikingly reduced by HCTZ (Fig. 2b,c). These results are expressed graphically in Fig. 2e and f.

### Podocyte injury appeared in the early stage of SSHT

**Figure 3:**
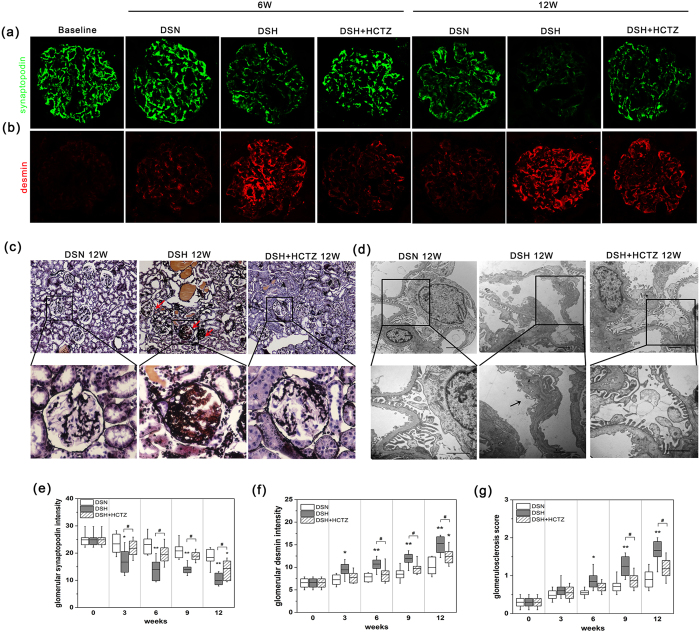
High salt loading induces glomerular injury with progressive glomerulosclerosis in DS rats. (**a**) Representative immunofluorescence images of glomerular synaptopodin staining in DS rats (magnification, 400X). (**b**) Representative immunofluorescence images of glomerular desmin staining in DS rats (magnification, 400X). (**c**) Representative light microscopy images (PASM) of glomeruli from 12 weeks; the DSH group exhibited serious glomerulosclerosis (arrowheads) compared with the DSN group, and HCTZ treatment decreased the appearance of glomerulosclerosis (magnification, 100X). (**d**) Transmission electron micrographs of glomeruli. Scale bar, 2 μm. (**e**,**f**) Chart of the quantification of the fluorescence staining intensity of glomerular synaptopodin and desmin. (**g**) Quantification of glomerulosclerosis at each time point. *p < 0.05, **p < 0.01 versus DSN group. ^#^p < 0.05, ^##^p < 0.01: DSH + HCTZ group versus DSH group.

DSH rats showed marked proteinuria (Fig. 1d), which could be associated with podocyte injury. We evaluated podocytes based on synaptopodin (a typical normal podocyte marker) and desmin (a conventional podocyte injury marker) expression by immunofluorescence^21^,^22^. Markedly decreased synaptopodin expression was observed in the DSH group compared with the DSN group (Fig. 3a), but treatment with HCTZ significantly reversed this change (Fig. 3a). The results are expressed graphically in Fig. 3e. Significantly enhanced desmin expression was observed in the DSH group compared with the DSN group (Fig. 3b), but HCTZ treatment markedly attenuated this alteration (Fig. 3b); these results are expressed graphically in Fig. 3f. Additionally, more severe focal-segmental or global glomerulosclerosis occurred in the DSH group compared with the DSN group (Fig. 3c); HCTZ treatment significantly reduced the glomerular damage in DSH rats (Fig. 3c), and these results are expressed graphically in Fig. 3g. We further assessed podocyte damage by transmission electron microscopy (Fig. 3d). The podocytes of DSH rats showed a lack of slit diaphragms and the appearance of foot process effacement and fusion at 12 weeks (Fig. 3d). On the other hand, HCTZ restored the podocyte injury towards normal in the glomerular of DSH group (Fig. 3d). These findings indicate that SSHT-induced podocyte dysfunction might be involved in the development of glomerulosclerosis, leading to tubulointerstitial fibrosis.

### PTC loss and the development of renal fibrosis

**Figure 4:**
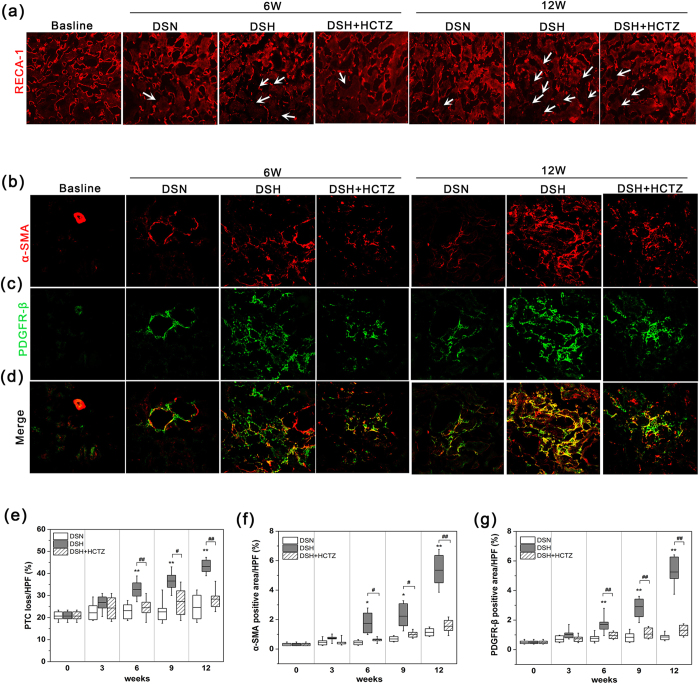
PTC loss and α-SMA accumulation in response to a high salt diet. (**a**) Representative images of RECA-labeled PTCs in the renal cortexes of each group; white arrows indicate the loss of PTCs (magnification, 200X). (**b**–**d**) Split panel confocal immunofluorescence images of myofibroblasts (red) and mesenchymal cells (green) during the progression of SSHT in DS rats. Composite images of α-SMA and PDGFR-β are shown at the bottom of the panel. Note that most interstitial cells in DS rats fed a high salt diet co-expressed α-SMA and PDGFR-β (magnification, 400X). (**e**) Graph of the change in the PTC index in the DSH, DSN, and DSH-HCTZ groups at each time point. (**f**,**g**) Graphs of the α-SMA- and PDGFR-β-positive areas in the long-term experiment in the DSN, DSH, and DSH + HCTZ groups. *p < 0.05, **p < 0.01 versus DSN group. ^#^p < 0.05, ^##^p < 0.01: DSH + HCTZ group versus DSH group.

PTC maintenance appears to be critical for preventing progressive renal fibrosis, and the loss of PTCs was previously demonstrated to play an essential role in impairing blood flow in the etiology of interstitial fibrosis^6^. Using RECA-1 as an endothelium marker to evaluate PTC density, greater PTC loss was observed at week 6 in DSH rats compared with DSN rats, and these alterations worsened over time (Fig. 4a). Meanwhile, PTC loss was significantly prevented by HCTZ treatment (Fig. 4a); these results are expressed graphically in Fig. 4e.

Because myofibroblasts are the prevailing cells that generate and deposit collagen I and III, we used immunofluorescence staining of α-SMA to assess myofibroblasts. α-SMA expression was upregulated after 6 weeks on the high salt diet (Fig. 4b). This change was aggravated over time with the progression of renal fibrosis, and the data are expressed graphically in Fig. 4f. Furthermore, we utilized immunofluorescence to evaluate the expression of PDGFRβ, which is a common marker of fibrosis-related mesenchymal cells, including pericytes, vascular smooth muscle cells, fibroblasts, and myofibroblasts; PDGFRβ expression indicates the proliferation of fibrosis-related cells. A significant increase in the PDGFRβ-positive area was observed in DSH rats at week 6 compared with DSN rats (Fig. 4c and g). Furthermore, PDGFRβ^+^ areas consistently expressed α-SMA (Fig. 4d). All these changes were significantly prevented by HCTZ (Fig. 4g).

### Apoptosis and cellular proliferation during the progression of SSHT

**Figure 5:**
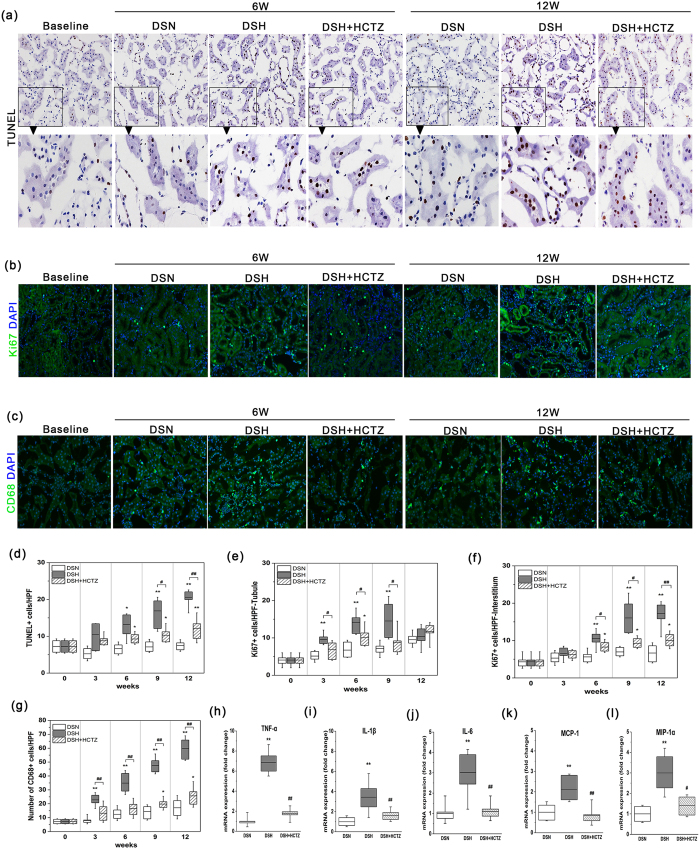
High salt diet induces apoptosis as well as renal tubule and interstitial cell proliferation in DS rats. Macrophage infiltration and inflammatory cytokine activation in DSH rats. (**a**) Representative images of TUNEL staining (brown staining) of the three groups at each time point. (magnification, 200X). (**b**) Immunostaining of Ki67 (green), which indicates renal cell proliferation, in each group (magnification, 200X). (**c**) Immunofluorescence images of CD68-labeled macrophages in each group at each time point (magnification, 200X). (**d**) Graph showing the number of TUNEL^+^ cells per HPF in the renal cortex in each group. (**e**,**f**) The chart depicting proliferating cells in the tubules and renal interstitium at each time point. (**g**) Morphometric quantification of the number of CD68^+^ cells (green) per HPF as an indicator of the degree of macrophage infiltration. (**h**–**l**) Quantitative RT-PCR was used to determine the mRNA levels of inflammatory cytokines and chemokines at the 12-week time point. Relative mRNA expression was determined after normalization to GAPDH, and the data are presented as the fold induction. *p < 0.05, **p < 0.01 versus DSN group. ^#^p < 0.05, ^##^p < 0.01: DSH + HCTZ group versus DSH group.

Cell cycle arrest and cellular apoptosis are two of the major epithelial mechanisms that contribute to chronic tubulointerstitial fibrosis^23^,^24^. Therefore, renal epithelial cell apoptosis and proliferation were assessed in the present study. The number of TUNEL-positive apoptotic cells significantly increased at week 6 in the DSH group. HCTZ administration markedly reduced the number of apoptotic cells (Fig. 5a and d). In addition, cell cycle labeling of kidney cells with the pan-cell cycle marker Ki67 showed that both interstitial cells and those of tubular origin were positive for Ki67 expression. As shown in Fig. 5b, proliferating cells were detected in the tubular epithelium and the interstitium. More Ki67^+^ cells were observed in the DSH group than in the DSN group (Fig. 5b and e). However, HCTZ treatment dramatically decreased the number of proliferating cells (Fig. 5b and e).

### Macrophage recruitment during the progression of SSHT

The roles of monocytes/macrophages in the development of tissue fibrosis have been increasingly recognized^15^. Macrophage infiltration significantly increased in the renal interstitium in DSH rats, and this alteration was clearly prevented by HCTZ treatment (Fig. 5c and g). Activated macrophages can release several inflammatory cytokines, such as tumor necrosis factor-α (TNF-α), interleukin (IL)-1β, IL-6, monocyte chemoattractant protein-1 (MCP-1), and macrophage inflammatory protein-1α (MIP-1α). The enhanced expression of these cytokines and chemokines in DSH rats was inhibited by HCTZ administration (Fig. 5h–l). We found that macrophage recruitment in DSH rats was associated with the upregulation of numerous cytokines and chemokines, which contribute to the pro-inflammatory microenvironment and the propagation of kidney injury.

### Activation of the Wnt/β-catenin signaling pathway and urinary Wnt4 expression in SSHT

**Figure 6:**
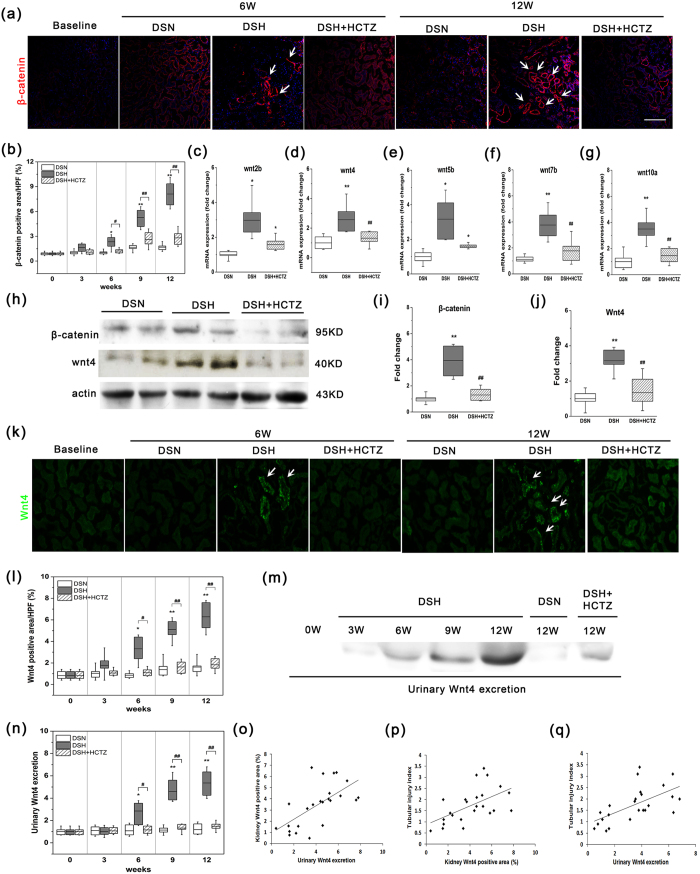
Wnt/β-catenin signaling activation and urinary Wnt4 excretion in DS rats fed a high salt diet. (**a**) Representative immunofluorescence micrographs of the time-dependent induction of β-catenin after high salt loading at various time points (magnification, 200X). Arrows indicate β-catenin-positive cells in the interstitium. (**b**) The chart of the quantification of β-catenin fluorescence intensity. (**c**–**g**) Quantitative RT-PCR was used to determine the mRNA levels of β-catenin signaling ligands at the time point with the highest β-catenin expression after high salt loading. Relative mRNA levels were determined after normalization to GAPDH, and the data are presented as the fold induction compared with the DSN group. (**h**) A dramatic increase in renal β-catenin and Wnt4 expression was observed by western blot analysis. (**i**–**j**) The quantitative data are presented, and relative β-catenin and Wnt4 protein levels (fold induction compared with the DSN group) are reported after normalization to β-actin. (**k**) Representative immunofluorescence micrographs of the time-dependent induction of Wnt4 after high salt loading in each group (magnification, 200X). (**l**) Quantification of immunofluorescence staining of Wnt4 in each group. (**m**) Representative western blot analyses of the dynamic changes in urinary Wnt4 expression. (**n**) Quantification of urinary Wnt4 excretion by western blot analysis in each group. (**o**) Correlation between kidney Wnt4 expression by immunofluorescence and urinary Wnt4 expression by western blot. (**p**) Relationship between kidney Wnt4 expression by immunofluorescence and tubular injury index. (**q**) Correlation between urinary Wnt4 expression by western blot and tubular injury index. The gels were run under the same experimental conditions. Cropped blots are shown (full-sized blots are presented in Supplementary Fig. S1). *p < 0.05, **p < 0.01 versus DSN group. ^#^p < 0.05, ^##^p < 0.01: DSH + HCTZ group versus DSH group.

Recent findings have indicated that Wnt protein ligands promote renal interstitial fibrosis by interacting with their receptors in the Wnt/β-catenin pathway^17^. Renal fibrosis is significantly associated with elevated β-catenin activity^25^. For these reasons, the role of the Wnt/β-catenin pathway in SSHT needs to be clarified. As shown in Fig. 6a, β-catenin protein levels markedly increased in DSH rats, and HCTZ administration decreased β-catenin expression (Fig. 6a,b). Next, we used real-time PCR (RT-PCR) to analyze the mRNA levels of Wnt ligands. As shown in Fig. 6c–g, the mRNA levels of Wnt2b, Wnt4, Wnt5b, Wnt7b, and Wnt10a were increased in the DSH group compared with the DSN group, whereas HCTZ administration decreased the overexpression of Wnt4, Wnt7b, and Wnt10a but did not significantly influence Wnt2b and Wnt5b expression. These results were confirmed by western blot analyses (Fig. 6h,i).

Our recent data demonstrated that increased Wnt4 expression is indicative of tubular injury^26^, while other reports have demonstrated interstitial and epithelial Wnt4 expression after chronic injury^20^,^27^. Enhanced renal Wnt4 expression was detected by western blot in the kidneys of DSH rats compared to those of DSN rats. However, this alteration was reversed by HCTZ treatment (Fig. 6h,j). These results were also confirmed by immunofluorescence (Fig. 6k,l). Furthermore, we tested whether Wnt4 could be detected in urine. Considerably increased urinary Wnt4 levels were observed in the DSH group compared to the DSN group (Fig. 6m,n), and HCTZ treatment notably diminished these changes (Fig. 6m,n). The excretion of urinary Wnt4 was positively correlated with kidney Wnt4 expression (r = 0.635, p = 0.001; Fig. 6o). Furthermore, both kidney Wnt4 expression (r = 0.55, p = 0.002; Fig. 6p) and urinary Wnt4 levels (r = 0.608, p = 0.002; Fig. 6q) were significantly associated with tubular injury. These data indicate that urinary Wnt4 could be used as a noninvasive biomarker for monitoring renal injury after HTN.

### Both renal and urinary Wnt4 are upregulated in HTN patients with tubular injury but normal estimated glomerular filtration rate (eGFR)

**Figure 7:**
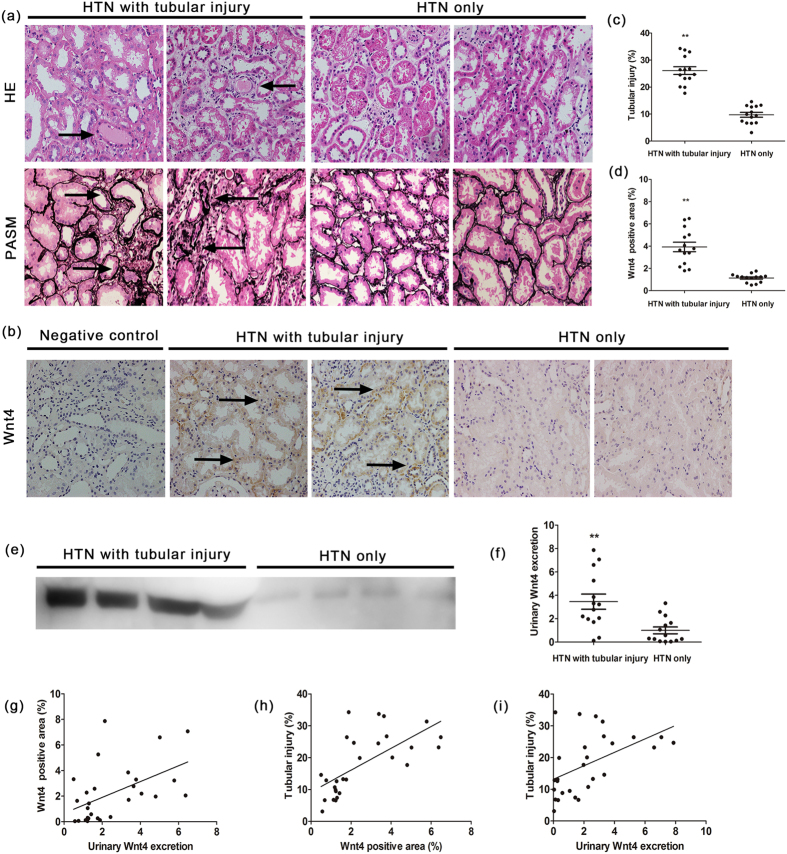
Upregulated renal Wnt4 and urinary Wnt4 excretion in HTN patients with tubular injury. (**a**) Representative of images from HE, PASM-staining sections of renal tissues in HTN patients with tubular injury and HTN only (magnification, 200x). (**b**) Immunohistochemistry of renal Wnt4 expression (magnification, 200X). (c) Kidney tubular injury score from HE staining. (**d**) Quantification of immunohistochemistry for renal Wnt4 expression.(**e**) Western blot expression of urinary Wnt4 excretion. Cropped blots are shown (full-sized blots are presented in Supplementary Fig. S1). (**f**) Quantification of urinary Wnt4 excretion from Western blot. (**g**) Correlation between renal Wnt4 expression and urinary Wnt4 excretion. (**h**) Correlation between renal Wnt4 expression and renal tubular injury. (**i**) Correlation between urinary Wnt4 excretion and renal tubular injury. *p < 0.05, **p < 0.01, versus HTN only.

We extended our observations from an animal model to patients to evaluate whether kidney Wnt4 expression was elevated in HTN patients with tubular injury. We enrolled HTN patients diagnosed by clinic manifestations and renal pathology who were divided into two groups: HTN without tubular injury (HTN only) and HTN with tubular injury. The clinical characteristics of the study cohort are shown in Table 1. Serum creatinine, 24-h urine protein, blood pressure, eGFR and other clinical characteristics had no significant differences between the two groups (Table 1). Representative images of hematoxylin-eosin (HE) and periodic acid-silver methenamine (PASM) staining of the two groups are presented in Fig. 7a. Tubular injury could be clearly observed in HTN patients with tubular injury (Fig. 7a,c). Enhanced Wnt4 expression was also evident in the renal tubules of the HTN with tubular injury group (Fig. 7b,d). Furthermore, urinary Wnt4 expression was significantly elevated in HTN patients with tubular injury compared with the HTN only group (Fig. 7e,f), consistent with the animal study. Additionally, there was a positive correlation between renal Wnt4 and urinary Wnt4 excretion (R = 0.505, P = 0.006, Fig. 7g). Both kidney Wnt4 expression (R = 0.661, P = 0.000) and urinary Wnt4 excretion (R = 0.501, P = 0.007) were positively correlated with renal tubular injury (Fig. 7h,i). These data suggest that urinary Wnt4 levels could be used as a noninvasive biomarker for monitoring tubular injury after HTN.

## Discussion

The present study provides novel insight into the mechanism underlying SSHT-induced renal injury. This study revealed that after the administration of an 8% high salt diet, DS rats developed aggravated renal interstitial fibrosis, tubular epithelial injury and glomerular damage over time. We further demonstrated that multiple mechanisms are involved in these alterations. DS rats fed a high salt diet resulted in obvious PTC loss, macrophage infiltration, apoptosis and cell proliferation following increased blood pressure. We also observed increased Wnt/β-catenin activation during the progression of SSHT. However, HCTZ administration attenuated these changes and decreased SBP. In addition, increased renal and urinary expression of Wnt4 was detected with time in the DSH group and was closely correlated with histopathological alterations. Furthermore, this alteration was also confirmed in a clinical study. Therefore, multi-target therapeutic strategies may be the most effective in preventing renal interstitial fibrosis in SSHT. Moreover, urinary Wnt4 levels may be a noninvasive biomarker for monitoring renal injury after HTN.

Salt plays an important role in the pathogenesis of HTN. It is known that SSHT in humans and several experimental animal models is associated with progressive kidney damage, leading to end-stage renal disease (ESRD). DS rats are an excellent model of SSHT and the associated kidney injury, as these animals exhibit many of the phenotypic characteristics of human HTN^28^. It has been known for several decades that the use of thiazide diuretics to treat HTN is beneficial in SSHT due to the ability of these drugs to promote urinary sodium excretion and decrease blood pressure^29^. In our study, HCTZ was administered to a treatment group to ameliorate SSHT and kidney injury. The noninvasive Doppler RI method was used to quantify alterations in renal hemodynamics and to measure the resistance of renal vessels. The increased renal RI may have been due to a combination of glomerular and tubulointerstitial lesions and renal fibrosis. Our RI data suggest a decrease in creatinine clearance and indicate significant renal damage in DSH rats. HCTZ treatment significantly reduced blood pressure by promoting salt excretion and lessening the volume, thereby reducing intrarenal resistance, improving cortical blood flow, and preserving renal function.

Although ischemic kidney injury is characterized by epithelial injury, our previous studies demonstrated that capillary loss typically precedes the development of prominent fibrosis^6^. To better understand the contribution of microvascular changes to renal fibrosis in the SSHT model, we performed immunofluorescence staining for RECA-1 and showed that the progression of SSHT was associated with PTC loss in areas of cortical tubular atrophy and interstitial fibrosis. This study also demonstrated that during SSHT injury, a marked loss of PTCs with only relatively mild renal injury occurred after 6 weeks in DSH rats. In addition, there was a progressive loss of PTCs in SSHT-induced renal injury, and a loss of identifiable PTCs was evident in the fibrotic areas, which may play an essential role in the process of renal interstitial fibrosis^30^. Previous studies have confirmed that activated fibroblasts change their phenotype and transform into myofibroblasts^31^. We used α-SMA as a marker of myofibroblasts, and interestingly, we discovered a population of PDGFR-β^+^ cells, of which the majority co-expressed α-SMA. Under normal conditions, PDGFR-β^+^ cells are located in the perivascular region. PDGFRβ^+^ cells may become partially activated and continue to proliferate in the interstitial region upon injury^32^,^33^. These results suggested that PTC damage activates PDGFR-β^+^ cells, which then proliferate and accumulate, thereby enhancing the subsequent progression of interstitial fibrosis.

Macrophages are widely recognized as contributors to the pathogenesis of renal fibrosis. Macrophages comprise heterogeneous populations of cells that belong to the mononuclear phagocyte system, and they have diverse roles in renal inflammation, the replacement of damaged and apoptotic cells, and remodeling^34^. Consistent with a previous report^35^, we observed that high salt loading significantly increased macrophage infiltration, which was associated with the upregulation of a multitude of inflammatory cytokines and chemokines, including TNF-α, IL-1β, IL-6, MCP-1, and MIP-1α. During the progression of renal fibrosis in the SSHT model, there was an increased presence of macrophages, which can release pro-inflammatory cytokines and chemokines. HCTZ administration significantly reversed kidney injury and renal interstitial fibrosis, coincident with decreased macrophage infiltration. Taken together, these data indicate that macrophage infiltration may play a role in SSHT-induced renal fibrosis, consistent with previous studies^35^.

The Wnt/β-catenin signaling pathway is conserved and regulates cell fate, function, and phenotype during kidney development^36^. Aberrant regulation of Wnt/β-catenin has been implicated in many kidney diseases^17^,^18^. Activation of the canonical Wnt pathway stimulates fibroblasts and induces fibrosis^37^, including renal fibrosis^38^. Inhibiting the Wnt/β-catenin pathway attenuates renal interstitial fibrosis^17^. A previous study confirmed that macrophages from the injured kidney are a source of increased canonical Wnt/β-catenin activity^13^. We demonstrated that Wnt/β-catenin accumulates after SSHT-induced kidney injury and confirmed that Wnt/β-catenin signaling is hyperactive and detrimental during the development of SSHT-induced renal interstitial fibrosis, consistent with our previous study used another hypertension animal model^39^. Furthermore, treatment with HCTZ significantly reduced Wnt/β-catenin activity and attenuated renal interstitial fibrosis. We further confirmed that several Wnt ligands were activated during SSHT-induced renal interstitial fibrosis, and these ligands significantly increased the expression of Wnt/β-catenin in tubules. Thus, these findings provide significant insight into the role of Wnt/β-catenin signaling in renal fibrosis and a new strategy for SSHT-induced renal fibrosis. Interestingly, urinary Wnt4 levels were upregulated with increased Wnt/β-catenin signaling in the SSHT model.

Our previous study had also confirmed that wnt/ß-catenin signaling had been activated in two-kidney one-clip Goldblatt mouse model, another HTN animal model^39^. Moreover, our and other studies had demonstrated that wnt/ß-catenin signaling contributed to renal interstitial fibrosis^17^,^25^,^40^. Therefore, wnt/ß-catenin signaling could be a common pathway to induced renal fibrosis following HTN. Previous studies had demonstrated that the DKK1, a Wnt antagonist, reduces wnt/β-catenin signaling accumulation and attenuates renal interstitial fibrosis^25^,^41^. It is likely that inhibiting wnt/β-catenin signaling will become a promising therapeutic target on renal injury and fibrosis induce by HTN.

In the SSHT model, we demonstrated that renal and urinary Wnt4 expression markedly increased in DSH rats at 6 weeks. We further confirmed that renal Wnt4 expression and urinary Wnt4 excretion were correlated with tubular injury in this model, consistent with our very recent study^26^. Kidneys are the target organs that are primarily injured by HTN, which is one of the leading causes of ESRD. However, serum creatinine is a late and poor indicator of acute kidney injury (AKI)^42^. A rise in serum creatinine is often a sign of severe kidney damage, even if the rise in creatinine is minimal, and therefore, is more indicative of dysfunction than damage. Tubular injury occurs in the initial phase of renal damage induced by HTN. Therefore, a novel biomarker for the early detection of tubular injury would be significant for early diagnosis and intervention. To further investigate this possibility, we extended our study to clinical patients. We enrolled 28 hypertensive patients with or without renal tubular injury who were diagnosed by renal biopsies and had normal serum creatinine and eGFR. Marked tubular injury, such as intratubular proteinaceous cast formation, tubular expansion and atrophy were observed in HTN patients with tubular injury compared with HTN-only group. Both kidney and urinary Wnt4 expression levels were upregulated in HTN patients with tubular injury accompanied by normal eGFR. It was encouraging that both increased kidney and urinary Wnt4 expression levels were significantly correlated with renal tubular injury, consistent with our recent study^26^. Thus, urinary Wnt4 levels may be a noninvasive biomarker for monitoring renal tubular injury after HTN. In our results, clinical characteristics were not significantly different between the two groups, but the HTN patients with tubular injury tended to have higher mean age and SBP. The lack of significant differences may be due to the limited number of patients. However, further studies and validation in larger cohorts are needed to confirm these findings. In addition, long-term follow-ups of clinical patients are needed to investigate whether urinary Wnt4 could serve as an efficacy biomarker to predict outcomes.

In conclusion, the present study provides novel insight into the mechanisms related to PTC loss and macrophage infiltration in SSHT-induced renal injury and fibrosis. Our findings suggest that Wnt/β-catenin signaling participates in these pathological processes. Therefore, multi-target therapeutic strategies may be the most effective in preventing renal interstitial fibrosis and preserving renal function in patients with SSHT. In particular, urinary Wnt4 may be a noninvasive biomarker for monitoring renal tubular injury after HTN.

## Materials and Methods

### Animals and protocol

Eight-week-old male DS rats were purchased from Charles River Laboratory (Beijing, China) and maintained on a diet of 0.3% NaCl for 1 week (n = 78). Six rats were sacrificed at baseline. The other 72 rats were divided into three groups: the high salt (DSH), normal salt (DSN), and DSH with hydrochlorothiazide (HCTZ) treatment (DSH + HCTZ) groups. Twenty-four rats were then switched to a diet of 8% NaCl (DSH). Twenty-four rats were maintained on 0.3% NaCl (DSN) as a normal control group. HCTZ (10 mg/kg/d) was orally administered to another 24 rats as previously reported^43^ when they were switched to the 8% NaCl diet (DSH+HCTZ), which was continued until the time of sacrifice. The rats in these three groups were sacrificed at weeks 3, 6, 9, and 12 (n = 6 per time point).

The study protocol was approved by the Ethics Committee of Harbin Medical University. The animal experiments were performed in strict accordance with the National Institutes of Health Guidelines for the Care and Use of Laboratory Animals. Rats were sacrificed under anesthesia (10% chloral hydrate, peritoneal injection), and all efforts were made to minimize discomfort and pain.

### SBP measurement and renal ultrasonography for blood flow and resistive index

SBP was measured using a tail cuff according to the manufacturer’s instructions (BP-98A, Softron, Japan). Measurements were obtained for each conscious rat every week until sacrifice. The rats were pre-warmed to 36 °C for 15–20 minutes in a bag before each measurement. The average of three pressure readings was recorded for each measurement. SBP was always measured at the same time of day (8–10 am).

Animals were anesthetized by a peritoneal injection of 10% chloral hydrate and placed supine on a heated table. Body temperature was maintained at 37.5 °C. After depilation, an acoustic gel (Hongxinyuan, China) was applied to the skin, and imaging was performed using Vivid 7 (GE, USA). A 12 MHz transducer was held immobile by an integrated rail system during imaging. All measurements were collected on the left side of the animal, including peak systolic and end-diastolic blood flow velocities (mm/sec) in the renal cortex in pulsed-wave Doppler mode. Cine loops were exported and analyzed to obtain the resistive index (RI).

### Sample collection and serum parameter measurements

DS rats were housed in an air conditioned room (22 ± 2 °C) under a 12-hour light-dark cycle and given free access to food and water. A 24-hour urine sample was collected weekly from individual rats in metabolic cages with free access to water but not food. The urinary protein content was determined using the nephelometry method (Siemens BN II, Deerfield, IL, USA) as previously described^22^. Urinary sodium and creatinine levels were determined using an automatic biochemistry analyzer (Cobas C311, Roche, Mannheim, Germany). Six rats from each group were sacrificed under anesthesia at weeks 0, 3, 6, 9, and 12. Serum samples were analyzed using an automatic biochemistry analyzer to assess serum albumin and creatinine levels.

### Clinical parameter measurements

All patients recruited in this study were admitted to the Nephrology Department of the second Affiliated Hospital of Harbin Medical University. Renal tissues of these patients were collected during renal biopsies, as all patients had persistent proteinuria and HTN. Urinary samples were collected from the patients in the morning of renal biopsy and stored at −20 °C within 2 hours of collection until serum creatinine and urinary Wnt4 were analyzed. HTN was defined as an average systolic blood pressure (SBP) ≥ 140 mmHg or an average diastolic blood pressure (DBP) ≥ 90 mmHg, or use of antihypertensive agents. All recruited patients recruited were diagnosed with primary HTN and excluded other primary and secondary renal diseases according to clinical characteristics and renal pathology. The diagnostic criteria of HTN with tubular injury were based on clinic characteristics and histopathology changes. The Internal Review Board of Second Affiliated Hospital of Harbin Medical University approved the study protocol, and all patients provided informed consent according to the latest version of the Helsinki Declaration on human research ethics. All methods were carried out in accordance with the approved guidelines.

### Histological studies by light microscopy

One-half of the right kidney was analyzed by light microscopy. The kidney samples were fixed in 10% neutral-buffered formalin for 12 hours, dehydrated in a graded ethanol series, embedded in paraffin for sectioning (2 μm) and stained with HE, Masson’s trichrome, Sirius red or PASM. The images were captured using a Nikon DS Ril (Tokyo, Japan). The HE-stained paraffin sections were analyzed using a blinded scoring method. For each square, the presence of tubule injuries, including intratubular proteinaceous casts, necrosis and apoptosis, was documented. The final score was presented as the percentage of positive squares. Interstitial fibrosis was quantified in Masson- and Sirius red-stained paraffin sections; images were captured in 10 random cortex fields per rat, and the mean area of positive staining was quantified using NIS-Elements software. The glomerulosclerosis score was semiquantitative, as previously described^7^.

### Immunostaining

Rat kidney tissues were subjected to immunofluorescence as previously described^6^,^13^; Ki67 staining of paraffin sections was performed by dewaxing, antigen retrieval, and antibody incubation. The following primary antibodies were used: anti-α-smooth muscle actin-Cy3 (1:200, Sigma-Aldrich, USA), rabbit anti-rat CD68 (1:200, Abcam, UK), mouse anti-rat RECA-1 (1:10, Abcam, UK), rabbit anti-rat PDGFRβ (1:100, Abcam, UK), rabbit anti-rat β-catenin (1:200, Abcam, UK), rabbit anti-Wnt4 (1:100, Santa Cruz Biotechnology, USA), rabbit anti-rat Ki67 (1:200, clone SP6; Thermo Scientific, Fremont, CA), mouse anti-rat desmin (1:200, Abcam, UK), and rabbit anti-rat synaptopodin (1:500, Abcam, UK). The following secondary antibodies were used: Alexa Fluor 594-conjugated donkey anti-mouse IgG (1:400, Jackson ImmunoResearch Laboratories, USA) and Alexa Fluor-488-conjugated goat anti-rabbit IgG (1:400, Jackson ImmunoResearch Laboratories, USA). The nuclei were stained with 4,6-diamidino-2-phenylindole (DAPI). PTC loss was determined based on anti-RECA-1 staining, images were captured at 200X magnification, and each image was split into 192 squares using a grid. CD68^+^, Ki67^+^, α-SMA^+^, PDGFRβ^+^, and β-catenin^+^ cells were quantified as previously described^39^. The fluorescent intensity of desmin and synaptopodin was measured by manually outlining the perimeters of ten glomeruli in each section and semi-quantifying the luminosity of the outlined regions. These images were obtained using a Nikon microscope (Nikon, Tokyo, Japan) and were processed using NIS-Elements software.

### Morphological studies by transmission electron microscopy

Renal cortex tissues (1 mm^3^) for transmission electron microscopy were fixed in cold 2.5% glutaraldehyde at 4 °C for 4 h. After three washes in 1 M phosphate buffer (pH 7.2), the tissues were fixed in 1% osmium tetroxide for 2 h, dehydrated in graded ethanol, and embedded in epoxy resin. Ultrathin sections (80–90 nm) were stained with uranyl acetate and lead citrate, examined, and photographed using a Hitachi 7650 transmission electron microscope (Tokyo, Japan).

### Immunohistochemical staining

As reported previously^26^, for human renal Wnt4 staining, 2-μm-thick deparaffin sections were incubated in 3% H_2_O_2_ for 10 min, and antigen retrieval was performed with citric acid buffer (pH 6.0) for 2 min in a pressure cooker while heating. The sections were supplemented with rabbit anti-rat Wnt-4 antibody (1:100, Santa Cruz Biotechnology, USA) and incubated overnight at 4 °C. Horseradish peroxidase-conjugated goat anti-rabbit IgG (BOSTER, Wuhan, China) was used as the secondary antibody, and coloration was performed using a DAB Kit (BOSTER, Wuhan, China).

### TUNEL staining

Terminal deoxynucleotidyl transferase-mediated dUTP nick-end labeling (TUNEL) staining was performed using an *in situ* cell death detection kit (Roche, Indianapolis IN, USA) according to the user manual. Briefly, the cryosections were incubated with the TUNEL reaction mixture for 1 hour at 37 °C in a humidified chamber. After washing with phosphate-buffered saline, the slides were incubated with peroxidase-conjugated antibody for 60 min at 37 °C. The sections were stained with DAB followed byhematoxylin, dehydrated and mounted. Apoptotic nuclei stained brown and were counted in ten randomly selected areas under 200X magnification.

### Quantitative real-time PCR analysis

Real-time PCR was used for the quantitative assessment of mRNA expression. Renal tissue was homogenized using TRIzol reagent (Invitrogen, China) to extract RNA. cDNA was synthesized using a high-capacity cDNA reverse transcription kit (Applied Biosystems, USA) according to the manufacturer’s protocol, and real-time PCR was performed using previously described methods^39^. Table 2 shows the utilized primer sequences. Three replicates per sample were assessed, the experimental threshold cycle (CT) values were normalized to those obtained for glyceraldehyde-3-phosphate dehydrogenase (GAPDH), and the fold differences in gene expression were determined using the 2^−ΔΔCT^ method.

### Western blot analysis

The preparation of kidney tissue homogenates and western blot analysis of protein expression were performed using routine procedures as described previously^22^. The following antibodies were used: rabbit anti-rat Wnt4 (1:200, Santa Cruz Biotechnology, USA), rabbit anti-rat β-catenin (1:5000, Abcam, UK), and anti-rabbit IgG HRP (1:5000; Abcam, UK). All of the western blot results were normalized to β-actin.

### Statistical analysis

All of the values are presented as the mean ± standard deviation (SD). The statistical analyses were conducted using one-way analysis of variance (ANOVA) with the Hochberg test and two-sample t tests, and correlations were determined by two-tailed Pearson correlation analysis in SPSS. P values less than 0.05 were considered significant. All statistical tests were conducted using SPSS 19.0 software (SPSS Inc., Chicago, IL, USA).

## Additional Information

**How to cite this article**: Wei, S.-Y. *et al*. Multiple Mechanisms are Involved in Salt-Sensitive Hypertension-Induced Renal Injury and Interstitial Fibrosis. *Sci. Rep.* **7**, 45952; doi: 10.1038/srep45952 (2017).

**Publisher's note:** Springer Nature remains neutral with regard to jurisdictional claims in published maps and institutional affiliations.

## Supplementary Material

Supplementary Information

## Figures and Tables

**Table 1:** Baseline characteristics of the enrolled patients.

	All patients	HTN with tubular injury	HTN only	P value
No. of patients included	28	14	14	
Age (years)	39.1 ± 27.3	42.9 ± 12.7	35.4 ± 11.3	0.110
Male/female ratio	18/10	11/3	7/7	0.115
Weight (kg)	74.5 ± 15.3	79.9 ± 13.9	69.1 ± 15.2	0.062
Blood pressure (mmHg)	142.5 ± 27.3/97.7 ± 17.4	161.4 ± 18.7/100 ± 22.5	152.5 ± 12.2/95.7 ± 10.9	0.149/0.493
24-h urine protein (g/d)	1.7 ± 1.3	1.9 ± 1.5	1.4 ± 1.0	0.321
Serum albumin (g/L)	42.7 ± 4.9	41.8 ± 5.2	43.5 ± 4.8	0.39
Serum creatinine (μ mol/L)	80.3 ± 15.4	84.5 ± 16.1	76.1 ± 14.0	0.151
eGFR (ml/min/1.73 m^2)^	111.9 ± 32.3	112.4 ± 30.6	111.4 ± 35.1	0.931

The chart includes the mean ± standard deviation of the physical and biochemical data of the HTN patients with and without tubular injury.

**Table 2:** Primers (rat) used in the experiment.

Studied gene		Sequence of oligonucleotides (5′-3′)
TNF-α	F	CAAGAGCCCTTGCCCTAAGG
	R	CGGACTCCGTGATGTCTAAGTACTT
IL-1β	F	TCAGGAAGGCAGTGTCACTCA
	R	CATCATCCCACGAGTCACAGA
IL-6	F	CTGATTGTATGAACAGCGATGATG
	R	GGTAGAAACGGAACTCCAGAAGAC
MCP-1	F	GGCCTGTTGTTCACAGTTGCT
	R	CCTGCTGCTGGTGATTCTCTT
MIP-1α	F	GCCTGCTGCTTCTCCTATGG
	R	TTGGACCCAGGTCTCTTTGG
Wnt2b	F	CAGGAGTGGTCCATGCTATCAC
	R	AGCGGACACCATAGTGGATGT
Wnt4	F	ACTGGACTCCCTGCCTGTCTT
	R	GTCCGGTCACAGCCACACTT
Wnt5b	F	GGGACCGTTTGAAAGAGAAGTATG
	R	TCATTTCGCAGGCAGTAGTCA
Wnt7b	F	TGCTTTGGCGTCCTCTACGT
	R	CTCCCCGATCACGATGATG
Wnt10a	F	TCCGACCTGGTCTACTTTGAGAA
	R	TCTGGCGCAGAATGTTGTGA
GAPDH	F	CGCATCTTCTTGTGCAGTG
	R	GAGGGTGCAGCGAACTTTATT

F = forward; R = reverse.
