# Supplementary material for: Multiple Mechanisms are Involved in Salt-Sensitive Hypertension-Induced Renal Injury and Interstitial Fibrosis
Source: Sci Rep. 2017 Apr 6;7:45952. doi: 10.1038/srep45952 (PMC5382679; doi:10.1038/srep45952)
Supplement: Supplementary Information [file srep45952-s1.pdf]

Multiple Mechanisms are Involved in Salt-Sensitive Hypertension-Induced Renal Injury and Interstitial Fibrosis

SHI-YAO WEI [1,a], YU-XIAO WANG [1,a], QING-FANG ZHANG[1], SHI-LEI ZHAO[1], TIAN-TIAN DIAO[1], JIAN-SI LI[1]., WEN-RUI QI[2], YI-XIN HE[1], XIN-YU GUO[1], MAN-ZHU ZHANG[1], JIAN-YU CHEN[1], XIAO-TING WANG[1], QIU-JU WEI[1], YU WANG[1], BING LI[1]*

[1]*Department of Nephrology, Second Affiliated Hospital of Harbin Medical University, Harbin, People's Republic of China*

[2]*Financial Mathematics, Beijing Normal University-Hong Kong Baptist University United International College*

[a] S.-Y.W. and Y.-X.W. contributed equally to this work.

Short title: Mechanisms of SSHT-Induced Renal Injury and Fibrosis

***Correspondence to:** Prof. Bing Li, Department of Nephrology, 2nd Affiliated Hospital of Harbin Medical University, 246 Xuefu Road, Nangang District, Harbin 150086, P.R.C., Email: icecreamlee@hotmail.com.

Key words: Salt-sensitive hypertension, renal interstitial fibrosis, Wnt/β-catenin signaling, Wnt4

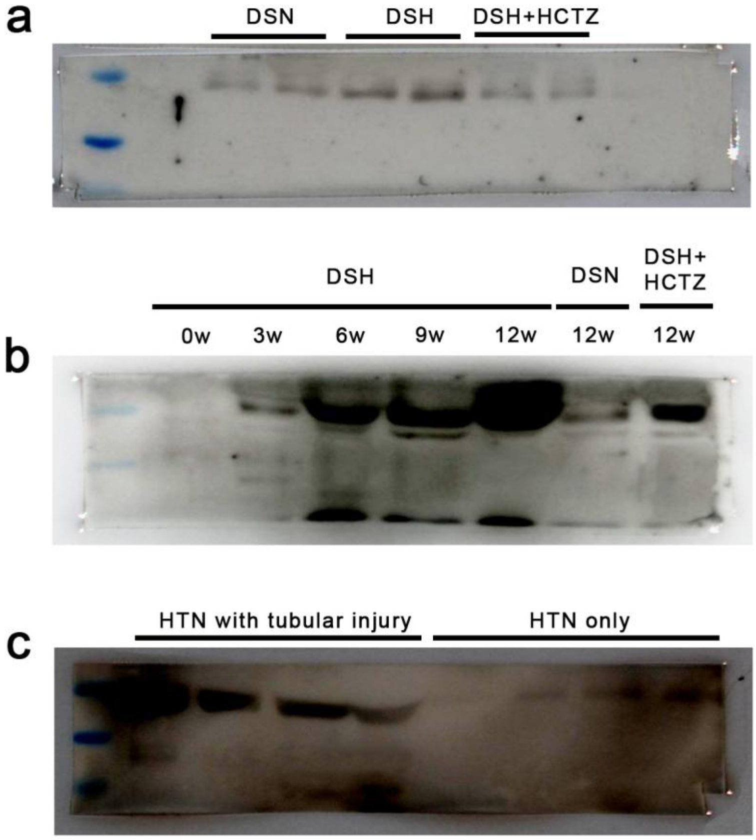

Supplementary Figure S1. Full-sized blots are shown. **a**. Kidney Wnt4 expression in DS rats at week twelve. **b**. Urinary Wnt4 expression in DS rats. **c.** Urinary Wnt4 expression in HTN patients.
